# Are urothelial carcinomas of the upper urinary tract a distinct entity from urothelial carcinomas of the urinary bladder? Behavior of urothelial carcinoma after radical surgery with respect to anatomical location: a case control study

**DOI:** 10.1186/s12885-015-1161-9

**Published:** 2015-03-18

**Authors:** Myong Kim, Chang Wook Jeong, Cheol Kwak, Hyeon Hoe Kim, Ja Hyeon Ku

**Affiliations:** Department of Urology, Seoul National University College of Medicine, 101 Daehak-ro, Jongno-gu, Seoul, 110-744 Korea

**Keywords:** Bladder cancer, Upper tract urothelial carcinoma, Radical cystectomy, Radical nephroureterectomy, Prognosis

## Abstract

**Background:**

To compare the prognosis of upper urinary tract (UUT)-urothelial carcinoma (UC) and UC of the bladder (UCB) by pathological staging in patients treated with radical surgeries.

**Methods:**

The study population comprised 335 and 302 consecutive radical surgery cases performed between 1991 and 2010 for UUT-UC and UCB, respectively. Five-year recurrence-free survival (RFS) and cancer-specific survival (CSS) rates were analyzed. The median follow-up period of all subjects was 59.3 months (range, 0.1–261.0 months).

**Results:**

No difference was observed in median patient age, distribution of pathologic T stage, or rates of positive surgical margin between the two groups. The UUT-UC group had significantly more frequent hydronephrosis than the USB group (48.1% vs. 20.2%, p < 0.001). However, the UUT-UC group showed significantly less frequent grade III tumors (28.1% vs. 58.6%, p < 0.001), lymphovascular invasion (18.8% vs. 35.8%, p < 0.001), and associated carcinoma *in situ* (9.0% vs. 21.9%, p < 0.001) than the UCB group. Five year RFS rates in the UUT-UC and UCB groups were 77.0% and 75.9%, respectively (p = 0.546). No significant difference in RFS rate was observed between pathological T stage subgroups. Five year CSS rates in the UUT-UC and UCB groups were 76.1% and 76.2%, respectively (p = 0.462). No significant difference was observed in CSS rate between the pathologic T stage subgroups.

**Conclusions:**

UUT-UC and UCB showed comparable prognosis at identical stages. However, our results should be verified in a prospective study due to the retrospective study design in this study.

## Background

Urothelial carcinoma (UC) is the fourth most common tumor in the United States and Europe, representing a heterogeneous groups of cancers [1]. UC can be located in any urothelial epithelia of the entire urinary tract. UC of the bladder (UCB) is the most common type of UC, accounting for 95%. Upper urinary tract (UUT)-UC represents 5% of UC at the initial diagnosis [2]. A 30–51% risk of bladder recurrence within 5 years was reported for patients who underwent radical nephroureterectomy for UUT-UC [3], with a 2–6% risk of developing a subsequent UUT-UC after UCB [4].

The two types of UC share common pathogenic mechanisms. They are expected to show analogous tumor characteristics [5] with similar prognostic risk factors [6,7]. However, although pathological staging of the two types of tumors is based identically on the natural anatomy of the UUT and the bladder, there have been some concerns that UUT may be more vulnerable to tumor spreading compared to that of the urinary bladder. The thinner muscle layer structure [8] and abundant lymphatic and blood channels [9] of the UUT are postulated to make tumor invasion and metastasis easier than those in UCB. In fact, it was reported UUT-UC was more invasive and metastatic than that of UCB at initial diagnosis [10], with 60% of UUT-UC as invasive at diagnosis compared to only 15% of UCB. There is strong clinical, etiological, epidemiological, and genetic evidence that UUT-UC and UCB should be considered distinct urothelial entities [11].

Currently, it is not clear whether the prognoses of these two types of UC are different for identical pathological staging. Therefore, we designed this study to compare the prognosis of UUT-UC and UCB by staging patients treated with radical surgery.

## Methods

### Patient selection

**Figure 1:**
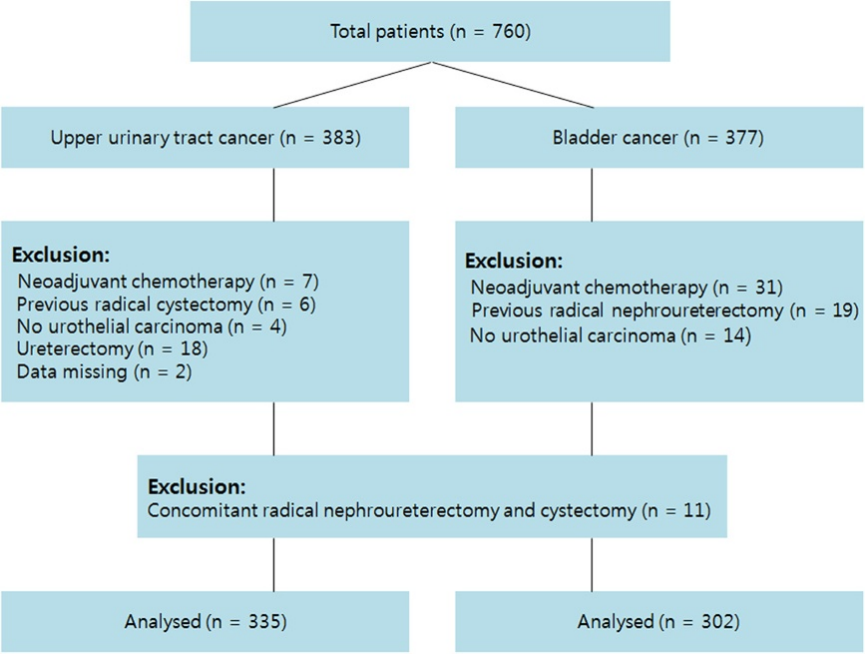
**Patient selection.**

This study protocol was approved by the institutional review board of Seoul National University Hospital, Seoul, South Korea. The study population comprised 760 consecutive radical surgery cases of UUT-UC or UCB performed between 1991 and 2010 at our institution (Figure 1). Of the 760 cases, 37 (9.7%) of radical nephroureterectomy cases and 64 (17.0%) of radical cystectomy cases were excluded from analysis. The reasons for exclusion are shown in Figure 1. Since there was a possibility of pathologic downstaging in patients who received neoadjuvant chemotherapy, we excluded 38 patients who received neoadjuvant. Thereafter, 11 (1.7%) of the remaining cases were identified to receive concomitant radical nephroureterectomy and radical cystectomy. Therefore, 335 patients with UUT-UC and 302 with UCB were analyzed in the current study.

### Treatments and follow-up

The workup, surgery, pathological review, and follow-up have been described in details previously [12,13]. Lymph node dissection (LND) was conducted in selective cases in the UUT-UC group that were suspicious for metastatic nodes based on preoperative evaluation. The extent of LND was decided by the surgeon. Radical cystectomy with pelvic LND was routinely performed in cases of muscle invasive UCB (pT ≥ 2), pT1 with concurrent *carcinoma in situ* (CIS), recurrence after intravesical Bacille Calmette-Guérin (BCG) immunotherapy, or with variant histologic subtypes such as micropapillary form. The extent of pelvis LND was limited below the bifurcation of common iliac vessels in most patients. A few patients underwent LND above the iliac bifurcation.

Excised specimens were processed according to standard pathological procedures. Tumor-node-metastasis staging of the tumor was classified by the 6th revised recommendation of the American Joint Cancer Committee 2002 [14]. Tumor grade was assessed based on the 1973 World Health Organization classification. Tumor recurrence was defined as local failure at the operative site, regional LNs, or distant metastasis at follow-up evaluations. Lymphovascular invasion (LVI) was defined as positive tumor cells in the vessel-like endothelium-lined space without the muscular wall. Cause of death was determined by the clinician based on chart review and authorized death certificate. Perioperative deaths occurring within 30 days of surgery were censored.

### Statistical analyses

Five-year recurrence-free survival (RFS) and cancer-specific survival (CSS) rates were analyzed. Kaplan–Meier curve and log-rank analyses were applied to compare survival in the two groups. The prognostic factors assessed were: tumor location (UUT vs. bladder), age, sex, body mass index (BMI), American Society of Anesthesiologists (ASA) score, presence of hydronephrosis, pathological T stage, pathological N stage, tumor grade, LVI or associated CIS, and margin status. All significant variables in the univariate analysis were included in a multivariate Cox model. Statistical analysis was performed using SPSS (SPSS Inc., Chicago, IL, USA). All tests were two-tailed with a significance level considered when p value was less than 0.05.

## Results

The descriptive characteristics of the 335 UUT-UC and 302 UCB patients are summarized in Table 1. The median follow-up for all subjects was 59.3 months (range, 0.1–261.0 months). Of the 302 UCB patients, 36 (10.5%) had no residual tumor (pT0). No difference in median age or pathologic T stage distribution was observed between the two groups. The two types of tumors were male dominant (79.1% vs. 89.4%, p < 0.001). The UUT-UC group had significantly higher BMI (24.2 vs. 23.4, p = 0.001), higher ASA score ≥ 2 (66.0% vs. 55.0%, p = 0.005), and more frequent hydronephrosis (48.1% vs. 20.2%, p < 0.001) than the UCB group. However, the UUT-UC group showed less frequent grade III tumors (28.1% vs. 58.6%, p < 0.001), LVI (18.8% vs. 35.8%, p < 0.001), and associated CIS (9.0% vs. 21.9%, p < 0.001) than the UCB group. There was no difference in the rate of positive surgical margins between the two groups (4.2% vs. 7.6%, p = 0.064).

**Table 1:** **Patient characteristics**

	Upper urinary tract cancer		Bladder cancer		P value
	No. of patients	%	No. of patients	%	
No. of patients	335	100	302	100	
Age, years					0.217
Mean	63.0		62.0		
Range	29.5-90.0		21.0-85.6		
Sex					<0.001
Male	265	79.1	270	89.4	
Female	70	20.9	32	10.6	
Body mass index, kg/m^2^					0.001
Mean	24.2		23.4		
Range	13.8-38.8		13.9-32.5		
ASA score					0.005
1	114	34.0	136	45.0	
≥2	221	66.0	166	55.0	
Hydronephrosis	161	48.1	61	20.2	<0.001
Pathological T category					0.584
≤pT1	131	39.1	113	37.4	
pT2	58	17.3	62	20.5	
≥pT3	146	43.6	127	42.1	
Pathological N category					<0.001
pN-	39	11.6	237	78.5	
pN+	16	4.8	65	21.5	
pNx	280	83.6	0	0.0	
Tumor grade					<0.001
≤II	241	71.9	125	41.4	
III	94	28.1	177	58.6	
LVI	63	18.8	108	35.8	<0.001
Associated CIS	30	9.0	66	21.9	<0.001
Positive surgical margin	14	4.2	23	7.6	0.064

*Abbreviations:* ASA = American Society of Anesthesiologists, LVI = Lymphovascular invasion, CIS = carcinoma in situ.

**Figure 2:**
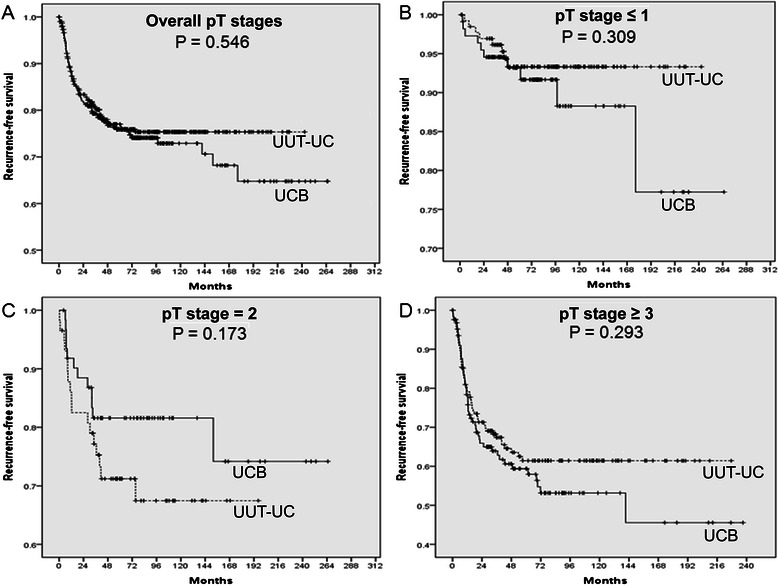
**Kaplan–Meier curves for recurrence-free survival (RFS) in patients with upper urinary tract urothelial carcinoma (UUT-UC) and urothelial carcinoma of bladder (UCB).** Between UUT-UC and UCB groups, no significant in 5 year-RFS rates were observed in **(A)** overall pathologic T stages (77.0% vs. 75.9%, p = 0.546), **(B)** pathological T stage ≤ 1 (93.3% vs. 93.2%, p = 0.309), **(C)** pathological T stage = 2 (71.2% vs. 81.6%, p = 0.173), and **(D)** pathological T stage ≥ 3 (61.4% vs. 59.4%, p = 0.293).

The Kaplan–Meier curves for RFS of the two groups stratified into three pathologic T stage subgroups are shown in Figure 2. Five year RFS rates of the UUT-UC and UCB groups were 77.0% and 75.9%, respectively (p = 0.546) (Figure 2A). No significant difference in RFS rate was observed among pathologic T stage subgroups (Figure 2B–D).

Univariate and multivariate analyses to predict RFS in all patients after radical surgery are summarized in Table 2. In the univariate analysis, BMI, presence of hydronephrosis, pathological T stage, pathological N stage, tumor grade, LVI, and positive surgical margin were highly significant predictors of recurrence. In the multivariate analysis including those parameters, pathological T stage (pT2, hazard ratio [HR]: 2.88, 95% confidence interval [CI]: 1.57–5.26, p = 0.001; ≥ pT3, HR: 4.68, 95% CI: 2.74–7.99, p < 0.001), pathological N stage (HR: 1.85, 95% CI: 1.18–2.89, p = 0.007), and LVI (HR: 1.50, 95% CI: 1.04–2.15, p = 0.029) remained independent predictors of recurrence. However, tumor location (UUT vs. bladder) did not affect RFS.

**Table 2:** **Univariate and multivariate Cox proportional hazard regression analyses of recurrence-free survival**

	Univariate		Multivariate	
	HR (95% CI)	P value	HR (95% CI)	P value
Tumor location				
Upper urinary tract vs. Bladder	0.906 (0.658-1.248)	0.546		
Age, years	1.016 (0.999-1.033)	0.057		
Sex				
Female vs. Male	1.098 (0.719-1.676)	0.665		
Body mass index, kg/m2	0.947 (0.898-0.999)	0.049	0.980 (0.929-1.033)	0.453
ASA score				
≥2 vs.1	0.927 (0.670-1.282)	0.645		
Hydronephrosis				
Present vs. Absent	1.609 (1.165-2.221)	0.004	1.378 (0.984-1.928)	0.062
Pathological T category				
pT2 vs. ≤pT1	3.588 (1.992-6.461)	<0.001	2.875 (1.573-5.256)	0.001
≥pT3 vs. ≤pT1	6.748 (4.087-11.141)	<0.001	4.675 (2.736-7.990)	<0.001
Pathological N category				
pNx vs. pN-	1.174 (0.810-1.700)	0.397	1.202 (0.805-1.794)	0.369
pN+ vs. pN-	3.232 (2.113-4.942)	<0.001	1.847 (1.180-2.889)	0.007
Tumor grade				
III vs. ≤II	1.955 (1.416-2.699)	<0.001	1.151 (0.802-1.652)	0.447
LVI				
Present vs. Absent	2.639 (1.911-3.645)	<0.001	1.496 (1.042-2.149)	0.029
Associated CIS				
Present vs. Absent	1.054 (0.681-1.631)	0.815		
Surgical margin				
Positive vs. Negative	2.777 (1.675-4.605)	<0.001	1.349 (0.797-2.282)	0.265

*Abbreviations:* HR = hazard ratio, CI = confidence interval, ASA = American Society of Anesthesiologists, LVI = Lymphovascular invasion, CIS = carcinoma in situ.

**Figure 3:**
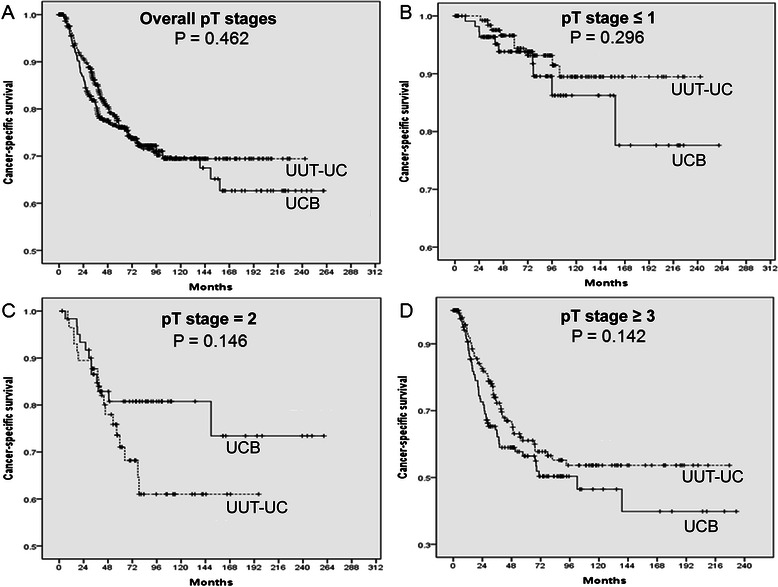
**Kaplan–Meier curves for cancer-specific survival (CSS) in patients with upper urinary tract urothelial carcinoma (UUT-UC) and urothelial carcinoma of bladder (UCB).** Between UUT-UC and UCB groups, no significant in 5 year-CSS rates were observed in **(A)** overall pathologic T stages (76.1% vs. 76.2%, p = 0.462), **(B)** pathological T stage ≤ 1 (94.4% vs. 93.8%, p = 0.296), **(C)** pathological T stage = 2 (71.0% vs. 80.8%, p = 0.146), and **(D)** pathological T stage ≥ 3 (61.1% vs. 56.4%, p = 0.142).

Five year CSS rates of the UUT-UC and BCB groups were 76.1% and 76.2%, respectively (p = 0.462) (Figure 3A). No significant difference in CSS rate was observed among pathologic T stage subgroups (Figure 3B–D).

Cox models used to predict CSS are shown in Table 3. In the univariate analysis, age, BMI, hydronephrosis, pathological T and N stage, tumor grade, LVI, and positive surgical margin were significant predictors of cancer-specific death. In the multivariate analysis, age (HR: 1.03, 95% CI: 1.01–1.044, p = 0.002), hydronephrosis (HR: 1.41, 95% CI: 1.02–1.96, p = 0.041), pathological T stage (pT2, HR: 2.71, 95% CI: 1.50–4.88, p = 0.001; ≥ pT3, HR: 4.96, 95% CI: 2.94–8.36, p < 0.001), pathological N stage (pN, HR: 1.99, 95% CI: 1.28–3.07, p = 0.002), and LVI (HR: 1.66, 95% CI: 1.16–2.37, p = 0.005) were independent predictors. Tumor location was not a predictor of CSS.

**Table 3:** **Univariate and multivariate Cox proportional hazard regression analyses of cancer-specific survival**

	Univariate		Multivariate	
	HR (95% CI)	P value	HR (95% CI)	P value
Tumor location				
Upper urinary tract vs. Bladder	0.889 (0.650-1.216)	0.462		
Age, years	1.022 (1.005-1.039)	0.010	1.027 (1.010-1.044)	0.002
Sex				
Female vs. Male	0.980 (0.643-1.493)	0.926		
Body mass index, kg/m2	0.921 (0.874-0.971)	0.002	0.965 (0.916-1.016)	0.177
ASA score				
≥2 vs.1	0.978 (0.711-1.343)	0.889		
Hydronephrosis				
Present vs. Absent	1.672 (1.221-2.289)	0.001	1.409 (1.015-1.957)	0.041
Pathological T category				
pT2 vs. ≤pT1	3.410 (1.919-6.058)	<0.001	2.707 (1.500-4.884)	0.001
≥pT3 vs. ≤pT1	7.115 (4.366-11.593)	<0.001	4.960 (2.943-8.359)	<0.001
Pathological N category				
pNx vs. pN-	1.126 (0.784-1.619)	0.520	1.126 (0.760-1.669)	0.554
pN+ vs. pN-	3.580 (2.364-5.422)	<0.001	1.986 (1.282-3.074)	0.002
Tumor grade				
III vs. ≤II	1.963 (1.432-2.689)	<0.001	1.105 (0.778-1.567)	0.578
LVI				
Present vs. Absent	2.875 (2.098-3.938)	<0.001	1.663 (1.164-2.374)	0.005
Associated CIS				
Present vs. Absent	0.971 (0.624-1.512)	0.898		
Surgical margin				
Positive vs. Negative	3.158 (1.930-5.166)	<0.001	1.483 (0.888-2.477)	0.132

*Abbreviations:* HR = hazard ratio, CI = confidence interval, ASA = American Society of Anesthesiologists, LVI = Lymphovascular invasion, CIS = carcinoma *in situ.*

## Discussion

UC can develop in a synchronous or metachronous multifocal manner at different urinary tract sites. Due to the relative preponderance of UCB, much of the clinical decision making regarding UUT-UC is extrapolated from evidence gained on UCB. However, because UUT-UC is biologically unique with appreciable genetic, molecular, and clinical differences from UDB [15], it remains questionable whether UCB findings could be safely extrapolated to UUT-UC.

Patients with UUT-UC generally have more advanced disease at the initial diagnosis [10,16]. Stewart et al. reported that tumor grade ≥ 3 (44% vs. 35%, p = 0.003) and pathologic T stage ≥ 2 (33% vs. 20%, p = 0.001) were more frequent in UUT-UC than those in UCB [16]. Several hypotheses have been proposed for the different tumor behavior of UUT-UC compared to UCB. Thinner muscle layer structure [8] and abundant lymphatic and blood channels [9] of UUT have been postulated to make tumor invasion or metastasis easier in patients with UUT-UC. These anatomical features of UUT representing thinner muscle/adventitia layer and smaller caliber lumen, can cause hardship to ensure sufficient healthy tissue for a safe surgical margin following conservative UUT-UC surgery [17]. Therefore, technical limitations of UUT-UC sampling compared to transurethral resection for bladder tumors may be the most important cause of staging differences between UUT-UC and UCB. Aside from these anatomical characteristics [8,9], some differences in the molecular biology of UUT have also been suggested as etiology of different tumor behavior [18-21]. Hartmann et al. reported that microsatellite instability (MSI) present in UTT-UC was correlated with mutation of human DNA mismatch repair genes and clinicopathological characteristic of tumor [18]. Roupret et al. also demonstrated that MSI was rarely encountered in UCB (approximately 3%), whereas it occurred in more than 15% of sporadic UTT-UC [19]. Catto et al. reported that the frequency of UUT-UC appeared to be significantly higher than UCB (94% vs. 76%) which might be associated with the poorer clinicopathologic outcomes of UUT-UC [20]. Single-nucleotide polymorphisms (SNP) variability of rs9642880[T] allele on 8q24 and rs798766[T] allele on 4p16 was not associated with disease aggressiveness of UCB. However, they were associated with more aggressive tumors when stratified by stage for UTT-UC [21]. These characteristics seem to make tumor invasion and metastasis easier in patients with UUT-UC [22]. Thus, radical nephroureterectomy with excision of the bladder cuff is recommended as the initial treatment of choice for high-grade UUT-UC.

However, it is still unclear whether the more aggressive behavior of UUT-UC have originated from different innate tumor biology or advanced status of the tumor at diagnosis. Some investigators have hypothesized that if the aggressiveness of UUT-UC is due to an initial higher stage, the prognosis may not be different between UUT-UC and UCB after stratification by stages. Catto et al. reported that 150 UUT-UC cases and 275 UCB cases showed similar prognoses (cancer-specific death, 35% vs. 43%) [17]. However, the population used in that study had a different distribution of pathological T stage (≥ pT2, 35% vs. 62%). Moreover, non-muscle invasive UCB cases received transurethral resection of bladder tumors, whereas all patients with UUT-UC underwent radical nephroureterectomy. Although the authors selected a subgroup of UCB patients with balanced pathological status to compare the prognosis between UUT-UC and UCB, the selection criteria were not described clearly. In addition, the selected cases were only from one institution of four participating centers. Therefore, there may have been selection bias. Moussa et al. [23] also found no difference in overall survival after controlling for the effects of tumor stage. In contrast to the study of Catto et al. [17], Moussa et al. [23] compared only patients who underwent radical surgery. They reported that patients with UCB have more advanced pathology than those of UUT-UC cohort.

In a recent multicenter study of 4,335 patients with UCB and 877 patients with UUT-UC, all patients were treated with radical surgery. It was found that stage and grade were independent predictors of CSS for the overall cohort [24]. However, in stage-specific analyses of patients with pT1 or less and pT4 disease, UCB and UUT-UC were independently predictive for CSS. They explained that the inferior outcomes of non-muscle invasive UCB patients as follows, “*Non-muscle invasive UCB patients undergo radical cystectomy because of features of aggressive biopsy. The lack of appropriate staging and grading, poor selection leads to high rates of radical nephroureterectomy for UUT-UC. Delay in diagnosis and/or treatment may differentially affect outcomes in these patients*”.

Our results are in consistent with those of previous studies [17,23]. Our subjects were comprised only of patients who received radical surgery for UUT-UC or UCB. As a result, our two groups showed similar pathological T stage characteristics (p = 0.584) (Table 1). Our data also revealed that UUT-UC and bladder cancer had identical 5-year RFS and CSS rates after radical surgery in all pathological T stage stratified subgroups (Figure 2 and Figure 3).

Our study had several limitations. This study was limited by the retrospective nature of the analysis with a relatively small number of patients. In addition, all patients with UCB underwent concomitant pelvic LND, whereas the UUT-UC cases underwent LND in only selective cases (55 of 280, 16.4%). Evidence of LND during radical nephroureterectomy is important for prognosis [25]. The low rate of LND in patients with UUT-UC might lead to an undefined bias regarding clinical outcomes. However, pelvic LND at the time of radical cystectomy is widely accepted, whereas LND at the time of radical nephroureterectomy is performed largely at the discretion of the surgeon, which may be due, in part, to the variable lymphatic drainage along the course of the ureter compared to the relatively confined lymphatic landing sites for the bladder [26]. Our results demonstrated that pNx was not an independent prognostic factor for RFS (p = 0.369) or CSS (p = 0.554) when compared to pN0 (Tables 2 and 3). Moreover, patients who received neoadjuvant chemotherapy were excluded from this study because the proportion of patients who received neoadjuvant chemotherapy was decisively different between the two groups. Because of the high resemblance of UUT-UC to UCB, neoadjuvant chemotherapy for UUT-UC is expected to produce similar results to those seen in UCB. However, one unique challenge for UUT-UC when incorporating neoadjuvant chemotherapy into therapy regimens is the associated limitation to clinical staging [27]. Since limited prospective data existed for neoadjuvant chemotherapy in UUT-UC, these data are currently insufficient to provide any recommendations. Recommended policies on neoadjuvant chemotherapy in UUT-UC and UCB are different [28-30]. Finally, there may have been dissimilarities in management between patients with UUT-UC and UCB in our cohort. Patients with pT1 or less UCB were generally treated with transurethral resection of the bladder tumor with or without intravesical therapy, whereas all patients with high-grade UUT-UC were recommended to undergo radical nephroureterectomy due to the inability to accurately stage and resect the tumor and/or effectively deliver intracavitary therapy [26].

## Conclusions

UUT-UC and UCB showed comparable prognosis at identical stages. However, due to the retrospective study design, our results should be verified in a prospective study.

## Abbreviations

UUT: Upper urinary tract
UC: Urothelial carcinoma
UCB: Urothelial carcinoma of the bladder
RFS: Recurrence-free survival
CSS: Cancer-specific survival
LND: Lymph node dissection
LVI: Lymphovascular invasion
BMI: Body mass index
ASA: AMERICAN Society of Anesthesiologists
CIS: Carcinoma *in situ*
